# Haemodynamic evaluation of alternative left ventricular endocardial pacing sites in clinical non-responders to cardiac resynchronisation therapy

**DOI:** 10.1007/s12471-015-0773-7

**Published:** 2015-12-08

**Authors:** B.M. van Gelder, R. Nathoe, F.A. Bracke

**Affiliations:** Department of Cardiology, Catharina Hospital, Michelangelolaan 2, 5623 EJ Eindhoven, The Netherlands

**Keywords:** Cardiac resynchronisation therapy, Non-responders, LV endocardial pacing, Haemodynamic evaluation

## Abstract

**Introduction:**

Non response to cardiac resynchronisation therapy (CRT) may be related to the position of the coronary sinus lead.

**Methods:**

We studied the acute haemodynamic response (AHR) from alternative left ventricular (LV) endocardial pacing sites in clinical non-responders to CRT. AHR and the interval from QRS onset to LV sensing (Q-LV interval) from four different endocardial pacing sites were evaluated in 24 clinical non-responders. A rise in LVdP/dtmax ≥ 15 % from baseline was considered a positive AHR. We also compared the AHR from endocardial with the corresponding epicardial lead position.

**Results:**

The implanted system showed an AHR ≥ 15 % in 5 patients. In 9 of the 19 remaining patients, AHR could be elevated to ≥ 15 % by endocardial LV pacing. The optimal endocardial pacing site was posterolateral. There was no significant difference in AHR between the epicardial and the corresponding endocardial position. The longest Q-LV interval corresponded with the best AHR in 12 out of the 14 patients with a positive AHR, with an average Q-LV/QRS width ratio of 90 %.

**Conclusions:**

Acute haemodynamic testing may indicate an alternative endocardial pacing site with a positive AHR in clinical non-responders. The Q-LV interval is a strongly correlated with the optimal endocardial pacing site. Endocardial pacing opposite epicardial sites does not result in a better AHR.

## Introduction

Cardiac resynchronisation therapy (CRT) has become an important treatment for patients with heart failure and left ventricular (LV) dyssynchrony [1–3]. However, clinical non-response to CRT is reported in 25–35 % of patients. Although a large variety of causes for a suboptimal response have been cited, most attention has been focused on the selection of patients eligible for CRT, and less on inadequate delivery of CRT therapy [4]. However, even with growing experience and improved materials and tools, the optimal position cannot always be reached in one of the tributaries of the coronary sinus. This can be due to the absence of suitable side branches in the posterolateral area, coronary vein stenosis, lead instability, high stimulation threshold, phrenic nerve stimulation, or a combination of the above [5–7].

We studied the acute haemodynamic response (AHR) of the implanted system and alternative left ventricular endocardial pacing sites in patients clinically not responding to CRT [8].

## Patients and methods

We asked patients who remained in New York Heart Failure class III or IV despite at least 6 months of CRT and adequate medical therapy and with no therapeutic options left to undergo an acute haemodynamic study in search of improving existing CRT, as previously described [8, 9]. Twenty-four non-consecutive patients, 23 males, age 72.8 ± 9.1 years, mean ejection fraction 22.5 ± 7.1 %, agreed to undergo this test (Table 1). The ECG prior to implantation showed left bundle branch block (LBBB) in 12, non-LBBB in 4, and right ventricular (RV) pacing in 8 patients.

**Table 1:** Characteristics of clinical non-responders (24 patients)

Patient	Gender	Age (Years)	NYHA Class	ICM/DCM	Ejection fraction (%)	QRS morphology	QRS width (ms)
Pt. 01	M	56	IV	DCM	13	LBBB	135
Pt. 02	M	78	III–IV	ICM	17	RVP	225
Pt. 03	M	75	III	ICM	12	LBBB	174
Pt. 04	M	70	III	ICM	19	RVP	196
Pt. 05	M	81	II–III	ICM	26	LBBB	165
Pt. 06	M	79	III	ICM	18	Non-LBBB	170
Pt. 07	M	74	III–IV	ICM	28	Non-LBBB	175
Pt. 08	M	87	III	ICM	42	LBBB	154
Pt. 09	M	79	III–IV	ICM	14	LBBB	209
Pt. 10	M	75	III	DCM	33	RVP	172
Pt. 11	M	55	III	DCM	30	LBBB	149
Pt. 12	M	81	III	ICM	14	LBBB	198
Pt. 13	F	72	III	DCM	23	RVP	152
Pt. 14	M	74	III	ICM	20	LBBB	152
Pt. 15	M	82	III–IV	DCM	25	RVP	200
Pt. 16	M	82	III–IV	DCM	25	Non-LBBB	175
Pt. 17	M	83	III	ICM	22	LBBB	192
Pt. 18	M	71	III	ICM	30	Non-LBBB	180
Pt. 19	M	71	III	ICM	17	RVP	166
Pt. 20	M	55	III–IV	ICM	20	LBBB	156
Pt. 21	M	66	III–IV	ICM	26	LBBB	174
Pt. 22	M	75	III–IV	ICM	22	LBBB	175
Pt. 23	M	61	III	ICM	27	RVP	160
Pt. 24	M	64	III–IV	ICM	17	RVP	210
Average	23M/1F	72.8 ± 9.1	3.2 ± 0.3	6DCM	22.5 ± 7.1	12LBBB/8RVP	175 ± 22

Patient characteristics of 24 clinical non-responders to CRT.

*NYHA class* New York Heart Association Class, *DCM* dilated cardiomyopathy, *ICM* ischaemic cardiomyopathy, *LBBB* left bundle branch block, *non*-*LBBB* non left bundle branch block, *pt*. patient, *RVP* right ventricular pacing.

In 23 patients, a 6F multipurpose angioplasty guiding catheter was introduced into the LV after a standard transseptal catheterisation. In one patient, the guiding catheter was introduced through the radial artery following coronary angiography. Through this guiding catheter, endocardial pacing and measurements of LVdP/dtmax were accomplished by roving a Medtronic 6416 temporary bipolar screw-in lead (Medtronic Inc. Minneapolis, MN.) and a RADI pressure wire (RADI Medical, a St Jude Medical Company, St. Paul, MN) inside the LV cavity.

Initially the atrioventricular and interventricular intervals of the implanted system were optimised to obtain the maximal LVdP/dtmax. LV pacing was performed from basal posterolateral, mid-posterolateral, LV apical and LV septal locations, and optimal AHR obtained after optimisation of the CRT system for all positions. If none of the 4 endocardial positions were opposite the position of the coronary sinus lead, an additional measurement was done at this location. A rise in LVdP/dtmax ≥ 15 % from baseline was considered to be a positive haemodynamic response.

In all patients with an AHR of ≥ 15 % during LV endocardial pacing, the interval between the onset of the QRS complex and the intrinsic activation at the LV electrode (Q-LV interval) was measured and the ratio between Q-LV/QRS width interval calculated. This ratio expresses the relation between Q-LV interval and QRS width, which is a better indicator for late or early LV sensing than the absolute value of Q-LV.

## Results

### Acute haemodynamic response of the total study population

**Fig. 1:**
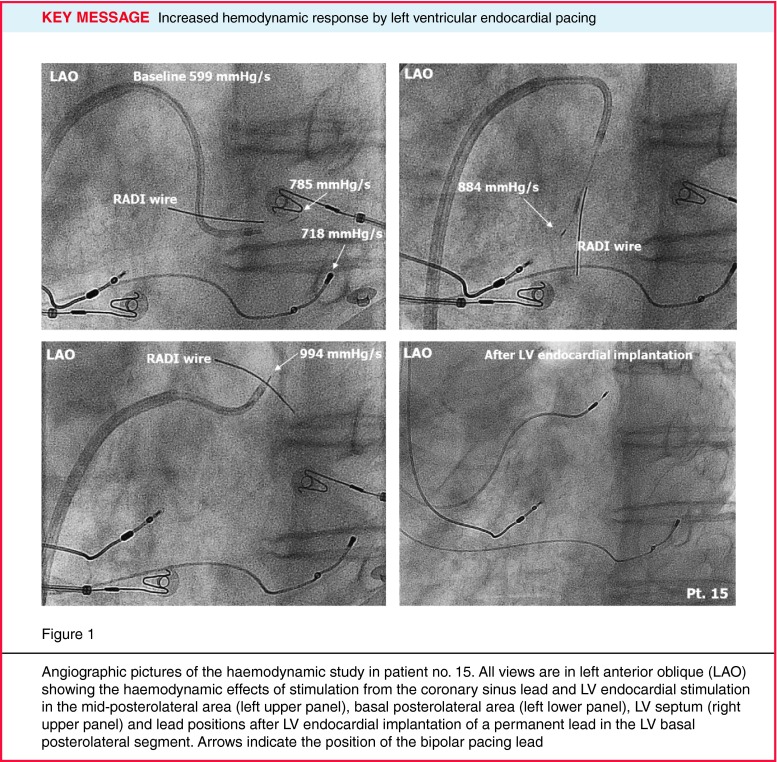
Angiographic pictures of the haemodynamic study in patient no. 15. All views are in *left* anterior oblique (*LAO*) showing the haemodynamic effects of stimulation from the coronary sinus lead and LV endocardial stimulation in the mid-posterolateral area (*left upper* panel), basal posterolateral area (*left lower* panel), LV septum (*right upper* panel) and lead positions after LV endocardial implantation of a permanent lead in the LV basal posterolateral segment. Arrows indicate the position of the bipolar pacing lead

Notwithstanding that all patients were clinical non-responders, in 5 patients LVdP/dtmax with the implanted coronary sinus system increased ≥ 15 % after optimisation: average 30.2 %, 15.6–44.5 % (Table 2). In 3 out of these 5 patients with the coronary sinus lead in a posterolateral position, endocardial pacing did not increase the LVdP/dtmax substantially (AHR less than 3 % and even an adverse effect from LV endocardial pacing was observed in one patient). In one of the two remaining patients with an apical position of the coronary sinus lead, the AHR could be increased during LV endocardial pacing from 19.7–66 % and we considered the increase sufficient to justify the upgrade to LV endocardial pacing (Fig. 1).

**Table 2:** Individual haemodynamic results of all clinical non-responders (24 patients)

Patient	QRS morphology	Baseline LVdP/dtmax (mmHg/s)	Position CS lead	AHR CS lead (%)	AHR CS-level-endo(%)	LV endo optimal position	AHR LVendo (%)
Pt. 01	LBBB	1177	PL-mid	5.8	2.7	PL-bas	24.2
Pt. 02	RVP	780	PL-mid	4.0	4.0	PL-bas	11.4
Pt. 03	LBBB	659	Ant-lat	6.4	8.0	PL-bas	31.6
Pt. 04	RVP	949	Ant-lat	9.3	9.7	PL-mid	24.8
Pt. 05	LBBB	918	LV apical	10.0	12.7	PL-bas	20.1
Pt. 06	Non-LBBB	1259	LV apical	1.8	5.7	LV apical	5.7
Pt. 07	Non-LBBB	893	PL-mid	− 23.4	–	LV septal	− 21.5
Pt. 08	LBBB	827	PL-bas	44.9	51.0	PL-bas	51.0
Pt. 09	LBBB	1113	PL-bas	− 21.7	− 2.9	LV apical	1.7
Pt. 10	RVP	1378	PL-mid	3.5	4.6	PL-bas	9.8
Pt. 11	LBBB	986	PL-mid	7.3	2.9	PL-bas	10.9
Pt. 12	LBBB	1159	LV apical	14.9	16.6	PL-mid	19.0
Pt. 13	RVP	1126	PL-mid	11.6	16.6	PL-mid	16.6
Pt. 14	LBBB	1024	PL-mid	3.6	6.8	PL-mid	6.8
Pt. 15	RVP	599	LV apical	19.7	23.8	PL-bas	66.0
Pt. 16	Non-LBBB	790	LV apical	− 9.1	-0.6	PL-bas	25.8
Pt. 17	LBBB	784	PL-bas	2.7	9.4	PL-mid	9.9
Pt. 18	Non-LBBB	929	LV apical	8.2	4.8	LV apical	4.8
Pt. 19	RVP	807	LV apical	1.1	–	PL-mid	7.8
Pt. 20	LBBB	1028	PL-mid	15.6	16.7	PL-bas	19.7
Pt. 21	LBBB	475	PL-mid	46.7	34.9	PL-mid	34.9
Pt. 22	LBBB	799	LV apical	8.2	15.6	PL-bas	34.3
Pt. 23	RVP	439	LV apical	23.9	26.7	PL-mid	31.0
Pt. 24	RVP	489	LV apical	12.7	17.2	PL-mid	24.1
Average		891 ± 247		8.7 ± 15.8	10.5 ± 8.9		19.6 ± 17.5

Haemodynamic measurements of 24 clinical nonresponders to CRT.

*Baseline LVdP/dtmax* LVdP/dtmax with intrinsic rhythm or right ventricular pacing, *AHR CS lead* acute haemodynamic response from the coronary sinus (CS) lead expressed as percentage rise in LVdP/dtmax from baseline, *AHR CS level endo* acute haemodynamic response at an endocardial location opposite the CS lead, *LV endo optimal position* anatomic site with the highest AHR, *AHR LV endo* Acute haemodynamic response from the optimal endocardial position. Grey shaded patients have an AHR from the CS lead ώ 15 % and are considered haemodynamic responders, *PL-bas* basal posterolateral, *PL-mid* mid-posterolateral, *RVP* right ventricular pacing.

In the 19 patients with an AHR from the implanted system < 15 %, 9 patients had an increase in AHR above the 15 % limit. The results of the total group of 24 patients showed a positive response in 11 patients with an apical lead position in 7, anterolateral in 2 and mid-posterolateral in 2 patients.

A negative response of endocardial pacing was observed in 13 patients with a basal posterolateral lead position in 3, mid-posterolateral in 8 and apical in 2 patients.

### Non-responders with LBBB

**Fig. 2:**
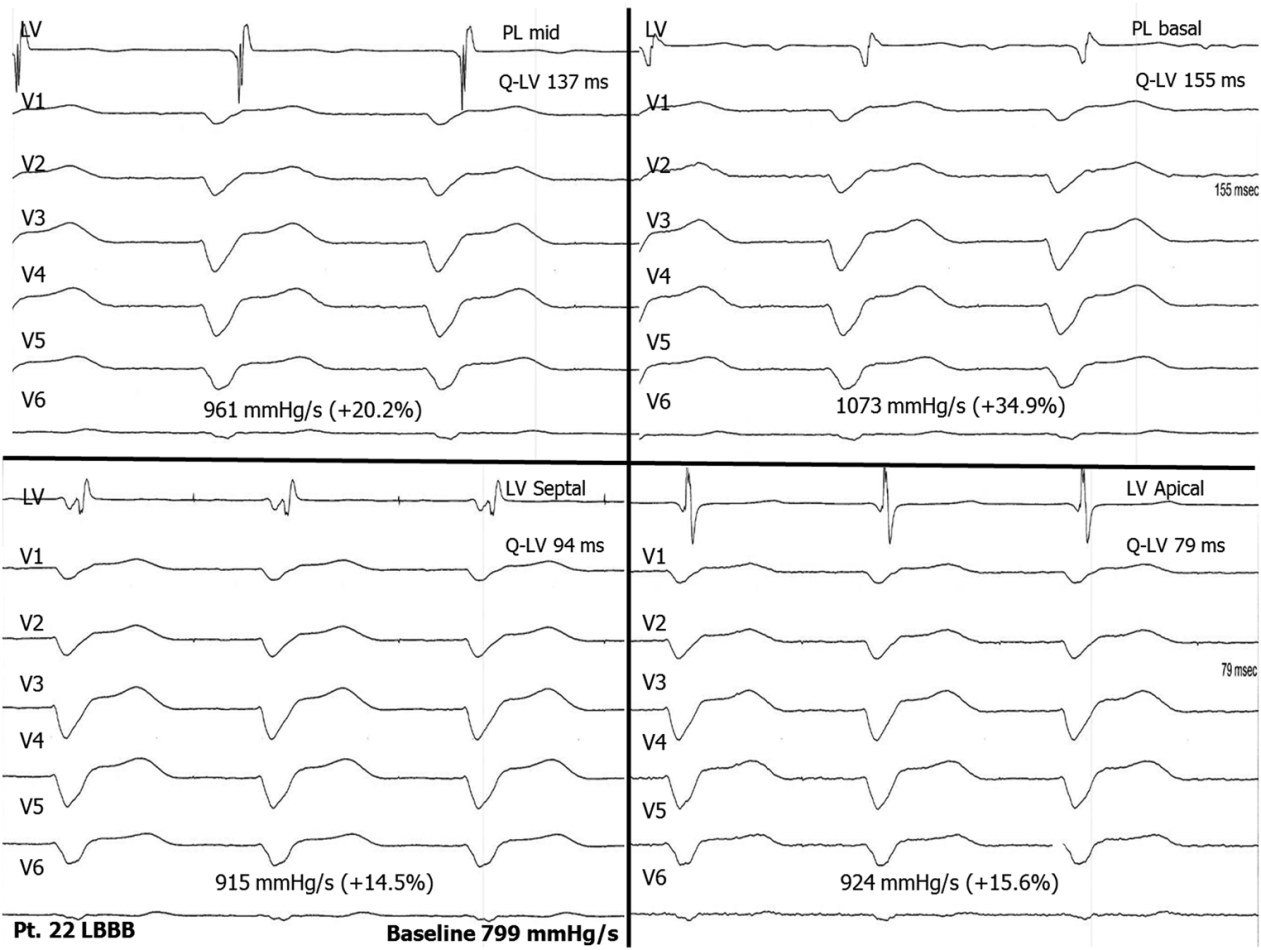
Recordings of the temporary study of patient no. 22 with a LBBB showing the haemodynamic effects and the timing of the LV endocardial electrogram from the different positions

In 5 out of the 9 acute haemodynamic non-responders (AHR < 15 %) with LBBB, the AHR could be increased above the 15 % level by LV endocardial pacing. The average AHR with the coronary sinus system was 9.6 % (5.8–14.9 %) and at the LV endocardial optimal position 25.8 % (19.0–34.3 %). The average QRS width in these patients was 169 ms (135–198 ms) and the average Q-LV at the optimal LV endocardial pacing site 157 ms (128–176 ms), resulting in a Q-LV/QRS ratio of 93 %. The position of the original coronary sinus lead was apical in 3, anterolateral in 1 and mid-posterolateral in 1 patient (Fig. 2).

In the remaining 4 patients, the average AHR at the optimal endocardial position increased < 15 %, with an average of 7.3 % (1.7–9.9 %). The average QRS width in these patients was 176 ms (149–209 ms) and the average Q-LV 136 ms (100–174 ms), resulting in a Q-LV/QRS ratio of 83 %. The coronary sinus lead position was posterolateral in all 4 patients.

### Non-responders with non-LBBB

**Fig. 3:**
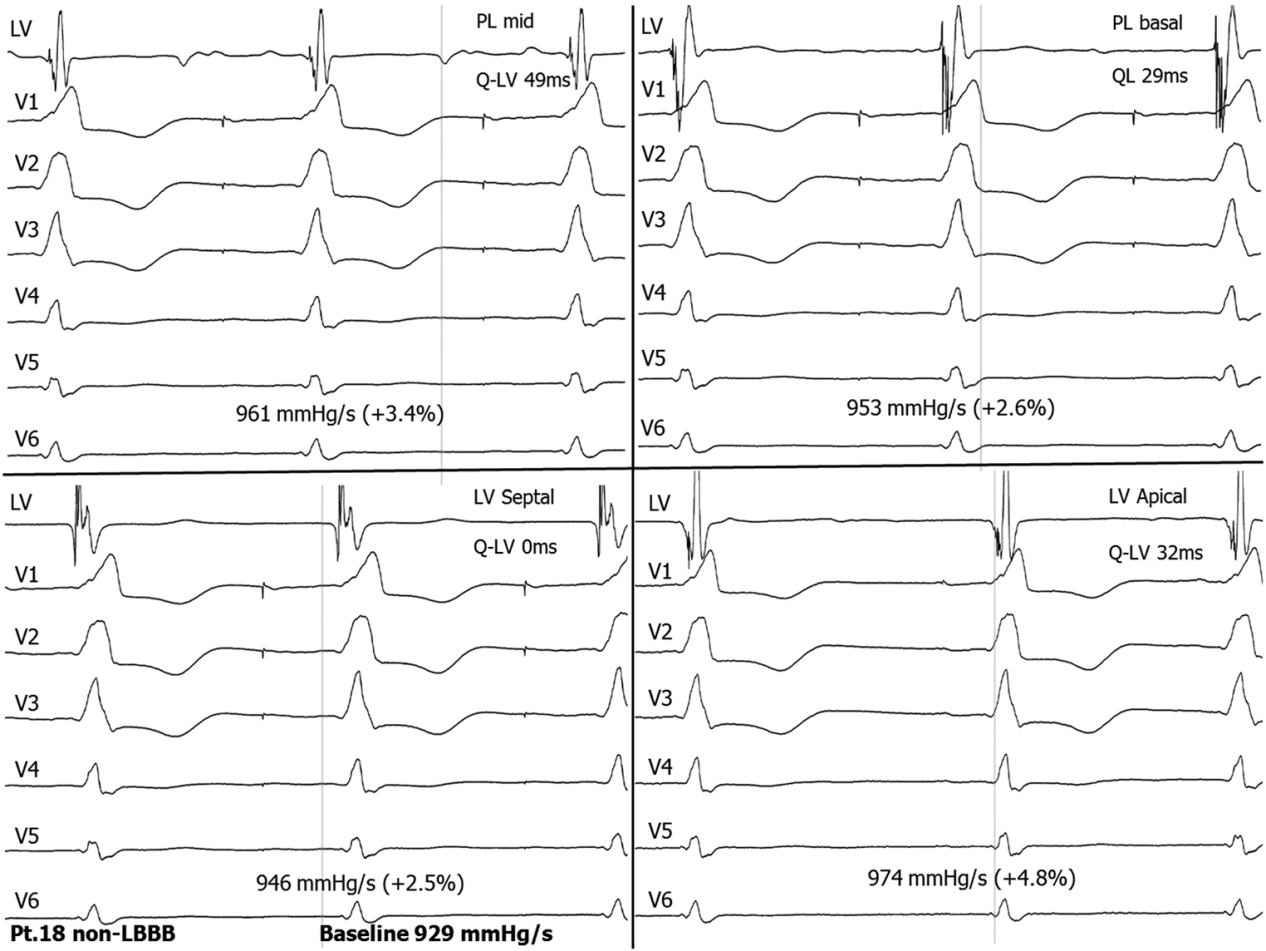
Recordings of a temporary study of patient no.18 with non-LBBB, showing the haemodynamic effects and the timing of the LV endocardial electrogram from the different positions. This recording illustrates that there is no conduction delay in the LV and a minimal haemodynamic effect

Four patients did not have LBBB. Of these, only one patient with RBBB, combined with left anterior hemiblock, showed an increase in the AHR, from − 9.1 % (apical coronary sinus position) to 25.8 % at an endocardial basal posterolateral position. Two of the other patients had an RBBB pattern (Fig. 3); one had a non-specific intraventricular conduction delay with a QRS complex of 175 ms. The coronary sinus lead position was mid-posterolateral in 2 and apical in 1 patient.

### Non-responders with right ventricular pacing

**Fig. 4:**
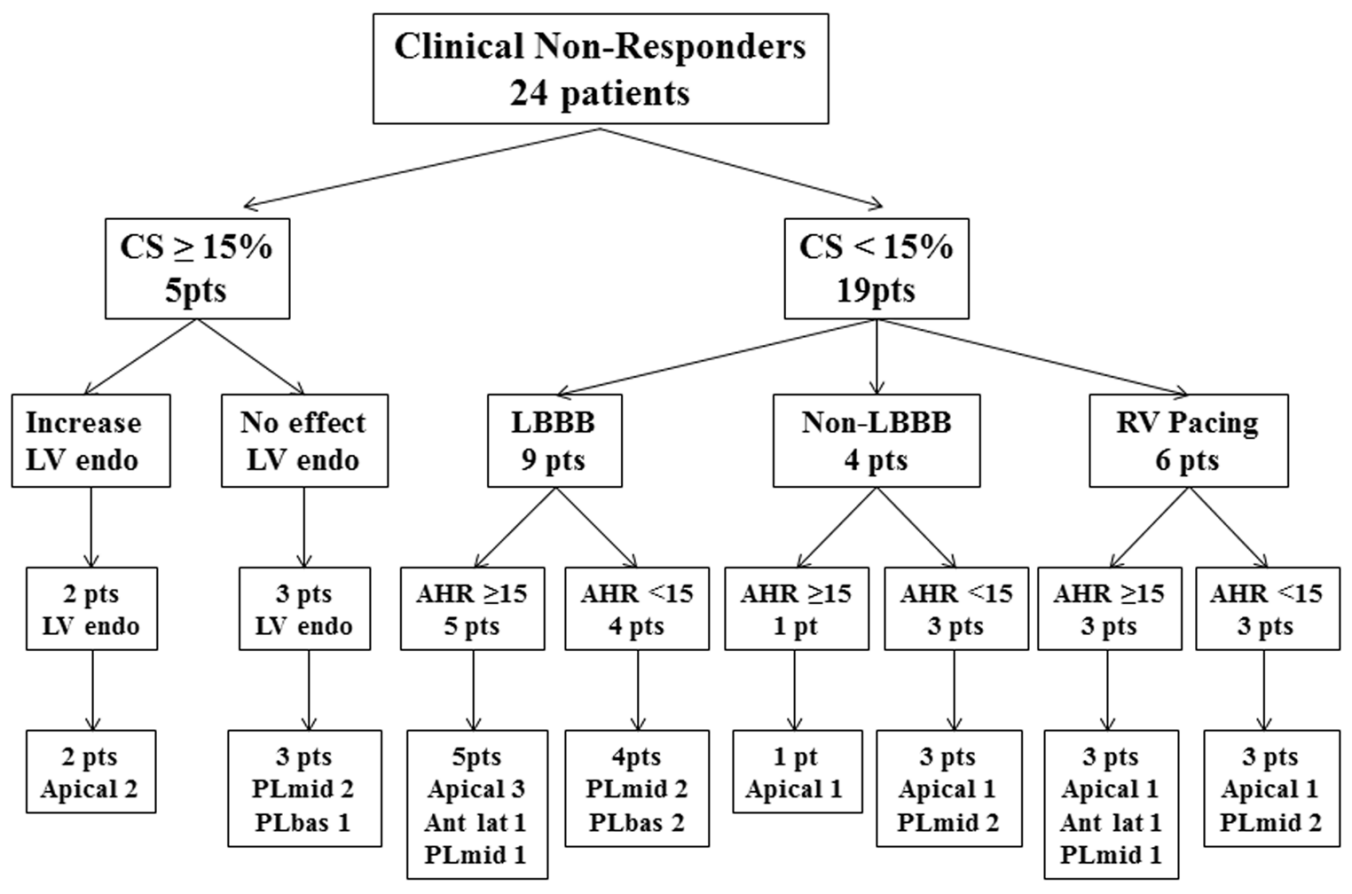
Flow chart of the study showing the haemodynamic results in relation to the coronary sinus lead positions. *AHR* acute haemodynamic response, *Ant lat* anterolateral, *bas* basal, *CS* coronary sinus, *endo* endocardial, *LBBB* left bundle branch block, *LV* left ventricular, *PL* posterolateral, *pts* patients, *RV* right ventricular

Three out of the 6 patients with RV pacing became haemodynamic responders (LV dP/dtmax from average 9.7 %, (9.3–12.7 %) increased to 21.8 % (16.6–24.8 %)). Coronary sinus leads were in an apical, anterolateral and mid-posterolateral position. In the other 3 patients the average AHR increased from 2.9 % (1.1–4.0 %) to 9.7 % (7.8–11.4 %). In 2 patients the coronary sinus lead was in a mid-posterolateral position, one in an apical position. Individual details are summarised in Table 2 and a flow chart overview in Fig. 4.

### Epicardial vs endocardial haemodynamics

When pacing endocardially, opposite the epicardial coronary sinus lead, we found that LVdP/dtmax did not differ significantly for any of the patients: LVdP/dtmax from the coronary sinus averaged 1046 ± 292 mmHg/s vs. 1068 ± 296 mmHg/s from the corresponding LV endocardial site (*p* = 0.37).

### Haemodynamics during endocardial pacing from different locations

We found that basal or mid-posterolateral segments had the highest AHR of the tested endocardial sites in all 14 patients who showed an AHR ≥ 15 % rise in LVdP/dtmax compared with baseline. Because either the basal or mid-posterolateral segment had the highest AHR, we also calculated the combined best results from basal posterolateral and mid-posterolateral (PL-best). PL-best had a rise to 30.2 ± 13.6 % in LVdP/dtmax, basal posterolateral 27.7 ± 15.2 %, mid-posterolateral 24.6 ± 12.6 %, LV apical 14.8 ± 9.1 % and LV septal 17.0 ± 11.2 %.

### Haemodynamic effect and Q-LV interval

We also found that in 12 out of the 14 patients (85 %) with an AHR ≥ 15 %, the LV endocardial site with the longest time interval between the onset of the QRS complex and LV sensing (Q-LV interval) resulted in the best haemodynamic response [10]. The average QRS width was 133 ± 22 ms (range 120–205 ms) and average Q-LV interval 155 ± 26 ms (128–205 ms). The average Q-LV/QRS width ratio, which is Q-LV expressed as a percentage of QRS width, was 90 %. The individual results of these data are summarised in Table 3.

**Table 3:** Haemodynamic results for all 4 endocardial locations and maximum Q-LV interval for 14 patients that showed a rise in LVdP/dtmax ≥ 15 % from endocardial pacing

Pts.	AHR PL-best (%)	AHR PL-basal (%)	AHR PL-mid (%)	AHR LV apical (%)	AHR LV septal (%)	QRS width (ms)	Max Q-LV Interval (ms)	Location Longest Q-LV
Pt.01	24.2	24.2	2.7	1.7	2.1	135	128	PL-bas
Pt.03	31.6	31.6	25.8	4.8	26.1	174	165	PL-bas
Pt.04	24.8	18.7	24.8	14.2	19.6	196	205	PL-mid
Pt.05	20.1	20.1	18.0	18.1	11.8	165	163	PL-mid
Pt.08	51.0	51.0	46.0	22.0	29.2	154	138	PL-bas
Pt.12	19.0	13.5	19.0	17.6	13.7	198	176	PL-mid
Pt.13	16.5	5.3	16.6	8.8	9.6	152	128	PL-mid
Pt.15	66.0	66.0	50.2	31.3	47.3	200	181	PL-mid
Pt.16	25.8	25.8	14.1	− 0.6	12.2	175	147	PL-bas
Pt.20	19.7	19.7	16.7	8.9	16.3	156	124	PL-mid
Pt.21	34.9	28.0	34.9	16.9	6.9	174	132	PL-mid
Pt.22	34.3	34.3	20.3	15.6	14.4	175	155	PL-bas
Pt.23	31.0	26.2	31.0	26.7	18.6	160	138	PL-mid
Pt.24	24.1	22.9	24.1	17.2	11.5	210	192	PL-mid
	30.2 ± 13.6	27.7 ± 15.2	24.6 ± 12.6	14.8 ± 9.1	17.1 ± 11.2	173 ± 22	155 ± 26	

*AHR* Acute haemodynamic response expressed as the percentage rise in LVdP/dt from baseline, *PL-best* best result from either basal posterolateral (PL-bas) or mid-posterolateral (PL-mid) region, *Max Q-LV* longest interval measured between onset of the QRS complex and intrinsic activation at the LV electrode. Location of this electrode is indicated in last column. Grey shaded area indicates 2 patient in whom the longest Q-LV interval did not correspond with the best haemodynamic response.

### Follow-up

Ten patients were considered possible candidates for LV endocardial pacing: 9 patients in whom a rise in LVdP/dtmax ≥ 15 % level was only obtained by endocardial pacing, and the patient that showed a substantial additional rise in AHR with LV pacing compared with coronary sinus pacing (patient 15). One patient died from progressive heart failure before the endocardial implant could be performed.

Of the remaining 9 patients, and after deliberation with the patients about the possible benefits and risks, 5 of them finally agreed to LV endocardial implant at the optimal LV site as indicated from the acute study [9]. All five patients improved clinically with a reduction of at least 1 class in the NYHA score after 6 months of follow-up. Two patients with a positive response in the temporary study refrained from implantation of an LV endocardial lead.

One patient with a positive AHR from endocardial pacing also improved significantly after changing stimulation from the distal to the proximal electrode with a more basal position, and became a clinical responder after optimisation of the atrioventricular and interventricular interval.

In one patient, the deterioration of his condition after initial improvement proved to be the result of a blunted chronotropic response after increasing beta-blocker dosage following an episode of atrial fibrillation resulting in an inappropriate ICD shock. Activation of sensor driven pacing restored his clinical status to the former level.

## Discussion

This observational study showed that endocardial LV pacing, guided by acute haemodynamic studies, could improve clinical outcome in patients not responding to conventional CRT. It also demonstrated the importance of lead location, as the clinical response improved with a posterolateral position of the lead instead of the original apical or anterolateral lead location. Conversely, none of the patients who already had a coronary sinus lead in a posterolateral position improved sufficiently with endocardial pacing at any site during the acute haemodynamic study to justify endocardial lead implant. This is in line with the MADIT-CRT trial, where the apical and anterior positions of the LV lead had a lower clinical response [10].

Noteworthy, and similar to what has previously been reported, endocardial pacing opposite the coronary sinus pacing site did not improve the AHR in our study [11–13]. This is in contrast with animal experiments that show significantly better haemodynamic results with endocardial vs. epicardial pacing at the same location [14]. There is no clear single cause for this difference and it is most probably related to variance in the anatomical and electrophysiological substrate. Besides this, in animal experience the lead configuration guarantees an exact position of the endocardial lead opposite to the epicardial position; this in contrast with human studies in which the endocardial lead is placed as close as possible to its epicardial counterpart.

Therefore, although not examined in this and other studies, one either might speculate that epicardial pacing opposite the optimal endocardial sites, via the coronary sinus or surgically by thoracoscopy, might have resulted in similar benefits. If feasible, this might be a good alternative to avoid the uncertainty of thromboembolic complications with LV endocardial pacing [15].

Our observational study also showed some limitations in the use of acute haemodynamic studies to predict clinical outcome. First, five clinical non-responders showed a clear AHR according to the accepted criteria (even with the higher than usual cut-off value of 15 % increase in LVdP/dtmax to exclude borderline cases) but failed to respond clinically [16].

Still, when a substantial further increase in LVdP/dtmax from endocardial pacing at a posterolateral position compared with the apical coronary sinus lead (66 vs. 19.7 %) was obtained in one patient, endocardial lead implantation resulted in a positive clinical response. This illustrates the limitation of cut-off values in CRT studies of the 15 % level as surrogate for differentiating between clinical response and non-response, and that what is considered a positive haemodynamic response can still be significantly improved in the presence of an anterior or apical lead position [10]. A similar discrepancy is found with echocardiography as the observed presence or absence of decrease in LV end-diastolic volume or end-systolic volume and clinical response does not always correlate [17].

To err on the safe side, we therefore proceeded to a permanent LV endocardial implantation when the final increase in AHR was at least 25 %. There was no clinical evidence for the choice of this percentage.

The best AHR correlates well with the longest time interval between onset QRS and LV sensing in relation to the QRS width in the individual patient [18].

The results of the group of patients with non-LBBB are in line with what could be expected according to the literature [19]. The absence of response correlated well to the lack of conduction delay in any of the LV segments on the intracardiac LV electrogram. The only responder to endocardial pacing in this group had a significant LV conduction delay due to the left anterior hemiblock that accompanied RBBB. It is therefore questionable if one should proceed with endocardial mapping in this cohort of patients given the disappointing results [19].

## Conclusions

This observational study showed that a temporary acute haemodynamic study could be useful in non-responders to establish the chance of improvement with alternative pacing sites before embarking on complex procedures as endocardial LV pacing. It remains, however, uncertain what the optimal cut-off value for increase in LVdP/dtmax is before the improvement justifies intervention. The longest Q-LV interval is a reliable indicator for the optimal electrode position in the individual patient.

Endocardial pacing opposite epicardial sites does not show acute haemodynamic improvement, so epicardial pacing at the optimal site may be a good alternative for endocardial LV lead placement. Although all patients who had a LV endocardial system implanted after a positive AHR improved clinically, larger randomised series are necessary to justify this technique before definitive conclusions can be drawn.

### Disclosures

B.M. van Gelder has received training and education on Pacing and CRT from St Jude Medical, is a consultant with RADI pressure wire systems, a St Jude Medical company, and consultant for Pacing and CRT with Sorin Group CRM SAS.

R. Nathoe, F.A. Bracke: None declared.
